# The Doctor as Artist, Connoisseur, and Collector

**DOI:** 10.1093/ofid/ofy013

**Published:** 2018-10-09

**Authors:** Shahram Khoshbin, Joel T Katz

**Affiliations:** 1 Department of Neurology, Brigham and Women’s Hospital, Harvard Medical School, Boston, Massachusetts; 2 Division of Infectious Diseases, Brigham and Women’s Hospital, Harvard Medical School, Boston, Massachusetts

**Figure 1.**
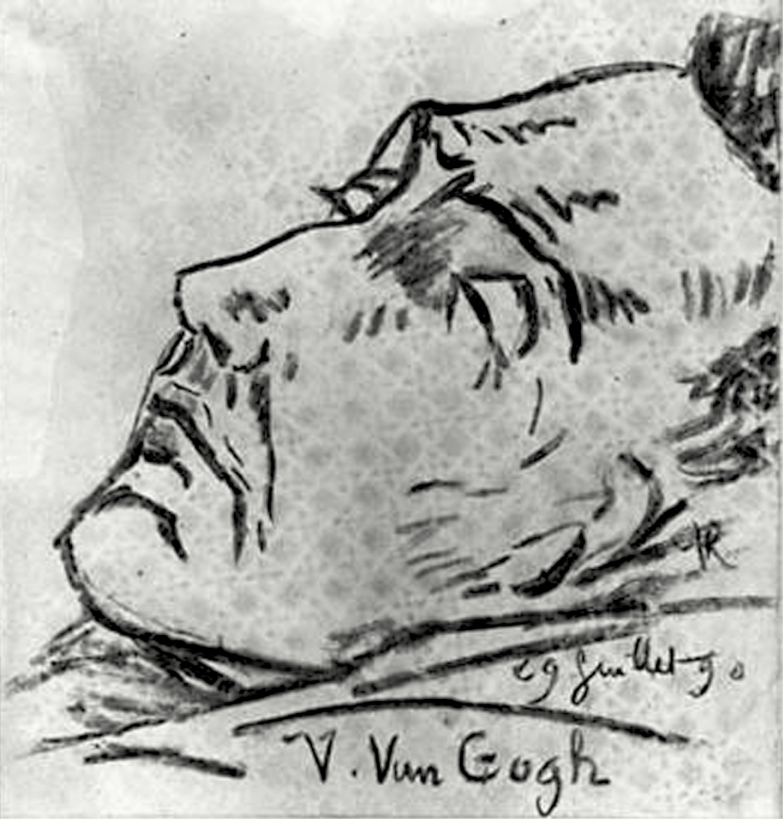
Vincent van Gogh on His Deathbed, by Paul-Ferdinand Gachet. July 29, 1890, Charcoal, Musee D’Orsay, Paris (https://realfrance.files.wordpress.com/2010/03/van-rijssel_vincent1890.jpg).

In July of 1890, in a letter describing the funeral of his friend, Vincent van Gogh, the famous impressionist Emile Bernard spoke of the person who delivered the eulogy, Dr. Gachet, and described him as a “great lover of art, who owns a great collections of paintings by impressionists, and he is an artist himself.” Bernard also mentioned that Gachet had been so overcome with grief that he could not deliver the eulogy properly, although he had known van Gogh for only a short period of time [1]. Although at the time Bernard was not aware it, van Gogh had painted portraits of his doctor, which eventually catapulted his doctor into the history of art. Of interest as well was that Gachet was present at van Gogh’s deathbed after van Gogh had shot himself, and Gachet made a drawing of the dying artist (Figure 1).

Paul Ferdinand Gachet (1828–1909) was born in Lille, France. He studied medicine first at the University of Paris. There he had been under the tutelage of Jean Pierre Falret and Jean-Etienne Esquirolle—both experts in diseases of the nervous system. Gachet then transferred to the Montplier Medical School, from which he graduated in 1858. He wrote his thesis on depression, “Etude sur la melancholie.”

As a physician, Gachet was quite eccentric. He started out in gynecology and pediatrics, worked as a doctor for the railroad, had personal tragedies (he lost his wife to “consumption,” probably tuberculosis), and raised 2 children. Both of his children kept his art collection, which they eventually gave to the French Republic. Gachet eventually moved to Auver sur-Oise in the suburbs of Paris, where he practiced general medicine. However, he was known to practice all sorts of alternative medicine, including palm reading. There, he made the acquaintance of many artists who visited the area. Some of them were his patients.

A relatively talented artist himself, Gachet’s interest in collecting art began when he got to know Gustave Corbet. Gachet eventually cared for and became friends with a panoply of other great artists, including Camille Pisaro and Paul Cezanne. Gachet met and knew Claude Monet and Auguste Renoir, as well as Victor Hugo. Gachet became van Gogh’s physician when, at the recommendation of Pisaro, van Gogh’s brother, Theo, arranged for Gachet to care for the ailing Vincent van Gogh, who had been diagnosed with epilepsy in the South of France. Because of Gachet’s training, and also probably because he himself was depressed, he diagnosed van Gogh as “melancholic.” It is interesting to note that the patient diagnosed the same in his doctor, and, in a letter, van Gogh commented: “But that when he talked of his collection, it brought a smile to his depressed face” [2]. Gachet had commented on other artists who exhibited symptoms of depression, including Cezanne and Daumier. Gachet considered depression as part and parcel of van Gogh’s genius. Gachet immediately became fascinated by van Gogh and showed van Gogh his vast collection of paintings and artifacts [3].

In the famous portrait of Dr. Gachet, the 2 striking features are Gachet’s face, which van Gogh himself describes in a letter: “I have done a portrait of Dr. Gachet with a melancholy face.” The other feature is the foxglove plant. Digitalis was at the time known as a treatment for depression. There is good indication that Dr. Gachet was using it himself. Because a side effect of digitalis is a yellow discoloration of vision, a theory has developed that the use of bright yellow colors by van Gogh was due to this treatment, although there is no documentation that van Gogh ever took digitalis.

**Figure 2.**
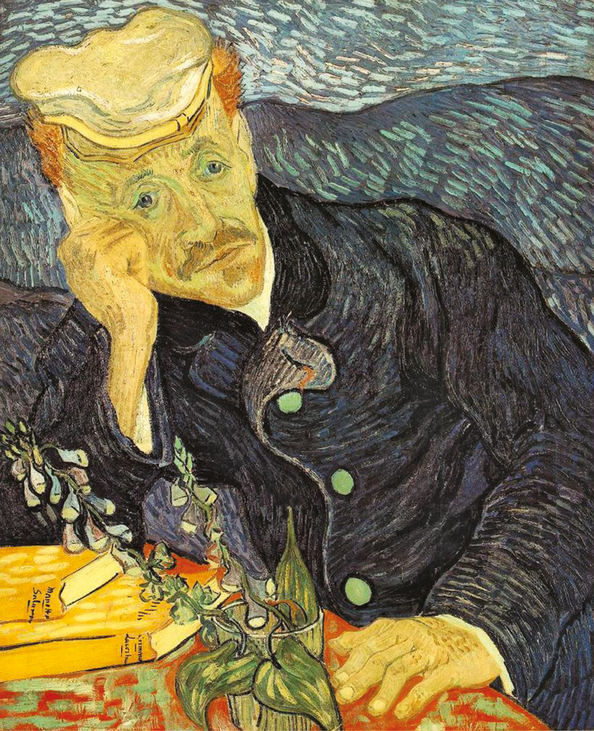
Portrait of Dr. Gachet (version 1), by Vincent van Gogh; June 1890; 67 × 56 cm; Private collection (https://en.wikipedia.org/wiki/Portrait_of_Dr._Gachet#/media/File:Portrait_of_Dr._Gachet.jpg).

**Figure 3.**
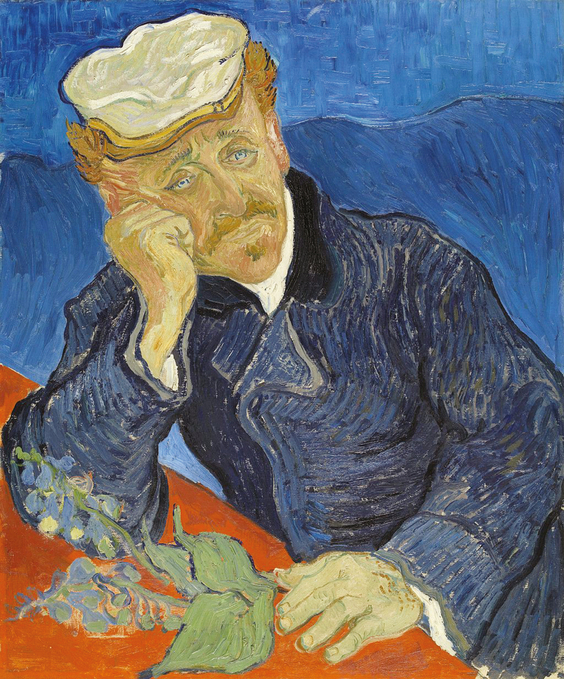
(cover) Portrait of Dr Gachet (version 2), by Vincent van Gogh. June 1890; 67 × 56 cm; Musee D’Orsay, Paris (https://en.wikipedia.org/wiki/Portrait_of_Dr._Gachet#/media/File:Vincent_van_Gogh_-_Dr_Paul_Gachet_-_Google_Art_Project.jpg).

**Figure 4.**
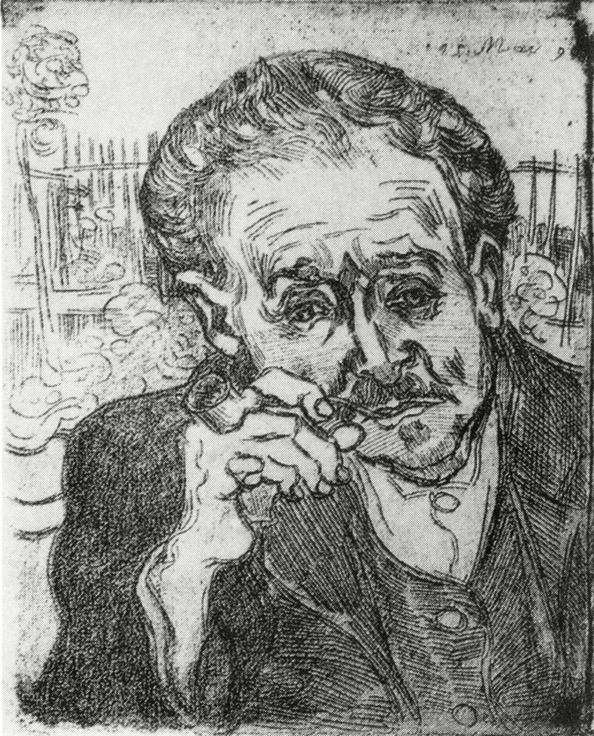
Man with Pipe (Portrait of Dr. Gachet), by Vincent van Gogh. May 1890 etching, 18 × 15 cm; 60 printings made (http://bostonartsdiary.com/wordpress/wp-content/uploads/2014/01/vanGogh_MFA_DrGachet_Etching_Detail_32.jpg).

The fate of that famous painting has been a mystery in itself. There are 2 versions (Figure 2 [version 1] and Figure 3 [version 2]). The first was sold by van Gogh’s family and eventually fell into the hands of the Nazis, and it was eventually purchased by a Japanese industrialist who kept it hidden for many years and was planning to cremate the painting along with his own body after his death. Instead, the painting was eventually sold to a private collector, and its fate is unknown. The second version was sold by Paul Gachet Fils to the French Republic and is in the Musee D’Orsay. Van Gogh also made etchings of Gachet using Gachet’s own printing press (Figure 4). A copy of this exists at the Museum of Fine Arts, Boston.
